# Persistence of Rabies Virus-Neutralizing Antibodies after Vaccination of Rural Population following Vampire Bat Rabies Outbreak in Brazil

**DOI:** 10.1371/journal.pntd.0004920

**Published:** 2016-09-21

**Authors:** Rita Medeiros, Viviane Jusot, Guy Houillon, Anvar Rasuli, Luzia Martorelli, Ana Paula Kataoka, Mohamed Ben Mechlia, Anne-Sophie Le Guern, Liliam Rodrigues, Rhomero Assef, Alvino Maestri, Reynaldo Lima, Yolande Rotivel, Valérie Bosch-Castells, Noël Tordo

**Affiliations:** 1 Universidade Federal do Pará e Instituto Evandro Chagas, Belém-Pará, Brasil; 2 Sanofi Pasteur, France; 3 Centro de Controle de Zoonoses, Sao-Paulo, Brasil; 4 Institut Pasteur, Paris, France; 5 Secretaria de Saude do Estado do Pará, Brasil; 6 Institut Pasteur de Guinée, Gamal Abdel Nasser University, Conakry, Guinea; Centers for Disease Control and Prevention, UNITED STATES

## Abstract

**Background:**

Animal control measures in Latin America have decreased the incidence of urban human rabies transmitted by dogs and cats; currently most cases of human rabies are transmitted by bats. In 2004–2005, rabies outbreaks in populations living in rural Brazil prompted widespread vaccination of exposed and at-risk populations. More than 3,500 inhabitants of Augusto Correa (Pará State) received either post-exposure (PEP) or pre-exposure (PrEP) prophylaxis. This study evaluated the persistence of rabies virus-neutralizing antibodies (RVNA) annually for 4 years post-vaccination. The aim was to evaluate the impact of rabies PrEP and PEP in a population at risk living in a rural setting to help improve management of vampire bat exposure and provide additional data on the need for booster vaccination against rabies.

**Methodology/Principal Findings:**

This prospective study was conducted in 2007 through 2009 in a population previously vaccinated in 2005; study participants were followed-up annually. An RVNA titer >0.5 International Units (IU)/mL was chosen as the threshold of seroconversion. Participants with titers ≤0.5 IU/mL or Equivalent Units (EU)/mL at enrollment or at subsequent annual visits received booster doses of purified Vero cell rabies vaccine (PVRV). Adherence of the participants from this Amazonian community to the study protocol was excellent, with 428 of the 509 (84%) who attended the first interview in 2007 returning for the final visit in 2009. The long-term RVNA persistence was good, with 85–88.0% of the non-boosted participants evaluated at each yearly follow-up visit remaining seroconverted. Similar RVNA persistence profiles were observed in participants originally given PEP or PrEP in 2005, and the GMT of the study population remained >1 IU/mL 4 years after vaccination. At the end of the study, 51 subjects (11.9% of the interviewed population) had received at least one dose of booster since their vaccination in 2005.

**Conclusions/Significance:**

This study and the events preceding it underscore the need for the health authorities in rabies enzootic countries to decide on the best strategies and timing for the introduction of routine rabies PrEP vaccination in affected areas.

## Author Summary

Animal control measures have decreased the incidence of human rabies in urban regions of Latin America. Currently, most cases of human rabies occur in rural areas and are transmitted by bats. In 2004–2005, rabies outbreaks affecting populations living in remote areas of Brazil prompted the widespread vaccination of exposed and at-risk populations. We evaluated the persistence of the humoral immune response for 4 years after vaccination in a rural population at daily risk of rabies exposure. Our aim was to evaluate the impact of vaccination in a rural setting to help improve management of vampire bat exposure. The participation of this Amazonian community was excellent, with 428 of the 509 (84%) who attended the first interview in 2007 returning for the final visit in 2009. The long-term RVNA persistence was good, with 85–88% of the participants evaluated at each yearly follow-up visit remaining seroconverted. Similar neutralizing antibody persistence levels were observed in participants originally given post-exposure or pre-exposure prophylaxis in 2005. This study and the events preceding it underscore the need for the health authorities in rabies enzootic countries to decide on the best strategies and timing for the introduction of routine rabies PrEP vaccination in affected areas.

## Introduction

Rabies is a viral zoonosis that affects mammals. It is caused by neurotropic viruses belonging to the family *Rhabdoviridae*, genus *Lyssavirus*. The International Committee on Taxonomy of Viruses (ICTV) recognizes today 14 species [1,2]; this taxonomy is rapidly evolving and the two more recently accepted Lyssaviruses isolated from a bat in Germany (Bokeloh bat lyssavirus) and from a civet in Africa (Ikoma lyssavirus) have been included as new species [3,4,5]. Most lyssavirus variants are found in bats and are known to cause rabies in humans and in domestic animals [6]. Interestingly, the isolates detected until now on the American continent all belong to the classical rabies virus (RABV), the species used in rabies vaccine. Lyssaviruses are neurotropic, causing acute encephalitis or “furious rabies” in about 70% of cases and a paralytic form of rabies in 30%. Not all exposures lead to illness, but once symptoms occur, rabies is almost always fatal. Therefore, proper prophylaxis to prevent infection must be administered promptly after exposure. Approximately 26,400 [95% confidence interval (CI) 15,200–45,200] human rabies deaths are estimated to occur worldwide each year using the “Cause of Death Ensemble” model, but the estimate rises to 61,000 (95% CI 37 000–86 000) when a probability decision-tree approach is used [7]. Rabies reservoirs and vectors include domestic as well as wild mammals, but human infection mostly results from bites from rabies-infected dogs. Animal control measures have decreased the incidence of urban human rabies transmitted by dogs and cats; and currently, in Latin American and Caribbean countries, most cases of human rabies are transmitted by bats [8,9].

The Pan American Health Organization (PAHO) implemented a multinational program against rabies in 1983, supporting intensive dog vaccination programs. The results have been very effective. In the Americas, canine cases decreased by 93% (from 15,686 to 1,131) and human cases decreased by 91% (from 355 to 35) between 1990 and 2003 [10]. In the countries where the circulation of canine rabies has been controlled, incidences of canine and human rabies continue to decrease in parallel, with 400 cases reported in dogs in 2010 and 10 in humans in 2012 in Latin America [11]. However, the number of human rabies cases caused by bats began to increase in Latin America in 2004, when more than half of the 87 reported cases were transmitted by vampire bats. Most cases were caused by outbreaks in Brazil (21 cases), Colombia (14 cases) and Peru (8 cases). In 2005, of the 60 reported cases of bat-transmitted human cases in Latin America, 42 were in Brazil and 7 in Peru (Amazonian area) [8]. Although human rabies cases have declined since 2006, cattle rabies in the region continues to increase, and a recent report from Peru estimated that the rabies seroprevalence in bats varied from 3 to 28% depending on the geographical region [12,13].

Although many human rabies outbreaks have been reported in northern Amazonian Brazil, few epidemiological studies have been performed. In 2004, a total of 21 people died during rabies outbreaks in two villages, Portel and Viseu, in the region of Pará State, Brazil, following bat bites (or as a result of bat rabies). In May 2005, 15 cases occurred in Augusto Correa, another rural municipality in the same region. These outbreaks, affecting populations living in remote areas, were of great concern to health authorities, prompting widespread vaccination of the exposed or at risk populations [14]. Following the rabies outbreak in 2005, more than 3,500 inhabitants of Augusto Correa received either post-exposure (PEP) or a pre-exposure (PrEP) prophylaxis. A few people were given booster vaccinations after possible rabies re-exposure, mostly following dog, bat, and monkey bites. As per national guidelines for PrEP in Brazil, if antibody titers < 0.5 IU/mL, the recommendation is to administer 1 booster dose via the IM route and to perform serological testing at D14. For re-exposed individuals who have previously received PEP no serological testing is done. Within 90 days of completing PEP, no vaccine is administered while within 90 days of incomplete PEP, the missing doses have to be given. More than 90 days after completing full PEP, 2 doses of vaccine (D0, D3) are recommended while if the PEP is incomplete, the recommendation is to administer the full 5-dose schedule based on the nature of rabies exposure [15].

## Methods

### Study design

This was a single-site, prospective epidemiological study designed to evaluate the persistence of RVNA in a population at risk of vampire bat rabies and who had previously received either PrEP or PEP regimens. The study also aimed at providing additional data on the need for booster vaccination against rabies. The results of 3 years of follow-up are presented here.

Outbreaks of human rabies cases occurred in 2004 and in 2005 in Augusto Correa, a rural municipality of approximately 27,000 inhabitants in Para state, northern Brazil. After the second outbreak, approximately 3,500 local residents of Augusto Correa were given either the standard five-dose intramuscular (IM) PEP (with or without rabies immunoglobulin administration) on Days 0, 3, 7, 14 and 28, or a three-dose PrEP vaccination series on Days 0, 7 and 21 or 28 with purified Vero cell rabies vaccine (PVRV, Verorab; Sanofi Pasteur, France).

This prospective study was conducted in 2007 through 2009 at the Arai health unit (USF Arai 3) in Augusto Correa, to evaluate the persistence of RVNAs in those who had been vaccinated in 2005. Each study participant was followed-up annually for 3 years (in 2007, 2008 and 2009). As recommended by WHO, an RVNA titer >0.5 International Units (IU)/mL was chosen as the threshold of seroconversion [16]. Participants with RVNA titers ≤0.5 IU/mL or Equivalent Units (EU)/mL at enrollment or at subsequent annual visits received booster doses of PVRV.

Anyone who had been vaccinated in 2005 was eligible to participate. Written informed consent was given by participants aged 18 years and above or by parents or legal guardians if younger. The study was conducted in accordance with the Edinburgh revision of the Declaration of Helsinki, International Conference on Harmonization (ICH) good clinical practice and applicable national and local requirements regarding ethical committee review.

The primary objective was to evaluate the persistence of RVNA following PrEP or PEP. Secondary objectives included describing RVNA titers following receipt of PVRV booster doses, estimating the incidence of clinical cases of rabies in the study population, and determining the correlation between the anti-rabies antibody titers obtained by the rapid fluorescent focus inhibition test (RFFIT) and a commercially available enzyme-linked immunosorbent assay (ELISA).

### Laboratory methods

Blood specimens (5 mL) were collected from each study participant at enrollment and at each of the three annual follow-up visits (when the patient came to the health center) for testing by RFFIT and enzyme-linked immunosorbent assay (ELISA). Blood serum specimens were divided into four 0.5 mL aliquots for testing. The RFFIT method was adapted from the original one [17] while changing both the cell line support (BHK21 cells instead of MNA cells) and the rabies virus strain used. RVNA titers of all specimens against the Pasteur virus strain PV (instead of the Challenge Virus Strain CVS) were measured by RFFIT at the Centro de Controle de Zoonoses (CCZ) laboratory in São Paulo, Brazil. Ten percent of those specimens were randomly selected for RFFIT re-testing at Institut Pasteur laboratory in Paris, France, using a vampire bat virus strain (instead of the CVS strain). In addition, the concentration of rabies virus anti-glycoprotein antibodies (EU/mL) in each blood sample was determined by ELISA (Pasteur virus strain) at Institut Pasteur laboratory in Paris, France, using the Bio-Rad Platelia assay as per the manufacturer’s instructions. The correlation between the RVNA titers measured by RFFIT and by fluorescent antibody virus neutralization (FAVN) assay (CVS in BHK21 cells) [18] was estimated at the CCZ laboratory, São Paulo, Brazil, using the specimens collected in 2007, the first year of the study.

### Statistical analysis

The immunogenicity analysis was descriptive; no hypotheses were tested. Seroconversion (RFFIT titer >0.5 IU/mL) rates and geometric mean antibody titers (GMTs) were calculated with their 95% confidence intervals (CIs). The sample size calculation was based on an expected seroconversion rate of 90% at 5 years after the primary vaccination series. A total of 140 subjects were required to ensure a 95% precision for a two-sided CI of 5%. Assuming 30% of the participants would be lost to follow-up at 5 years after primary vaccination, a total of 200 subjects had to be included. However, to anticipate additional dropouts, subgroup analyses and insufficient sera for laboratory testing, the planned enrollment was 500 participants. The study populations included in the analysis comprised: 1) all the evaluable study participants in each follow-up year, 2) participants who received a booster dose of vaccine at enrollment or during a follow up year, and 3) participants who did not receive booster doses of vaccine either at study entry or in any follow-up year. Missing data were not replaced.

The primary study endpoint was the number and percentage of subjects with RVNA titers >0.5 IU/mL each year using the RFFIT assay. We performed an analysis by gender and age group (i.e., 2–5, 6–17, 18–40, 41–60, and >60 years of age). The number and percentage of subjects with RVNA titers >0.5 EU/mL using the ELISA test was calculated in the overall study population for each of their follow up visits.

For inter-group comparisons, quantitative variables and ordinal qualitative variables were compared using Student’s *t*-test or ANOVA (parametric data) and the Wilcoxon or Kruskal–Wallis test (nonparametric data). Qualitative variables were compared using the Chi square test (or Fisher exact test when frequencies were less than five for at least one category). The correlations of GMTs measured by two different assays were determined by Pearson’s correlation coefficient (*r*). The correlations between percentages of participants with titers >0.5 IU/mL or EU/mL measured by RFFIT, FAVN or ELISA were calculated using the Kappa coefficient (κ).

## Results

### Study participants

**Fig 1:**
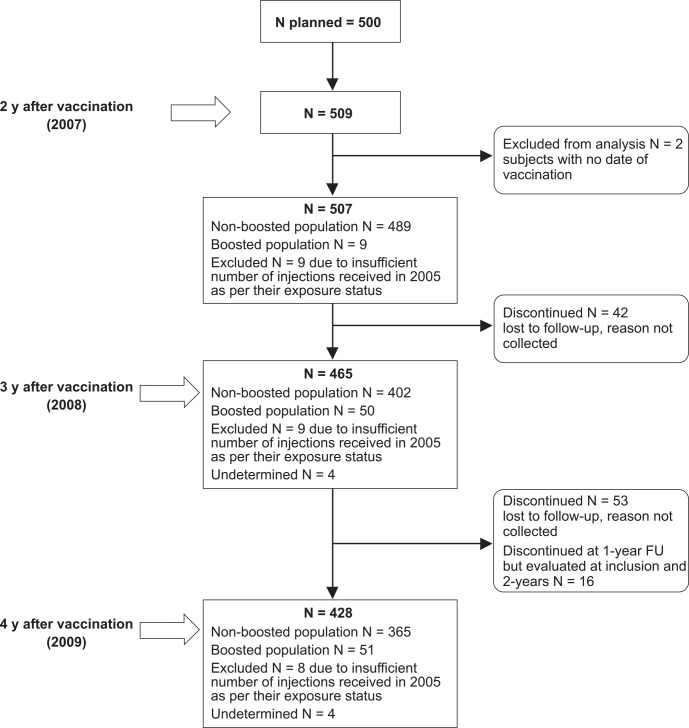
Participant disposition.

A total of 509 participants were enrolled in 2007 (Fig 1). Two of the 509 participants were excluded because their vaccination dates could not be confirmed. Among the 507 participants included, 496 (97.8%) were immunized (either PEP or PrEP) in 2005, eight in 2004 and three in 2006. In 2008, 42 participants were discontinued and 465 (91.7%) returned for evaluation. In 2009, 53 of the 465 remaining participants were discontinued and 16 of the 42 subjects who had been discontinued in 2008 came back so that a total of 428 (84.4%) participants were present in 2009. The mean (±SD) follow-up duration was 22.6 ± 6.9 months (range 0.0–26.5 months).

Among the 95 participants who did not complete the study or did not attend all of the visits, 91 (95.8%) were lost to follow-up and four died (one following an epileptic coma and three of different cancers). In 2007, nine participants were excluded from analysis because they had not received a complete PEP schedule (i.e., <5 vaccine doses). Four additional participants were excluded from analysis in 2008 because of missing data (no booster dose information). One subject who had previously been excluded from analysis in 2008 withdrew from the study in 2009.

### Participant demographics and rabies vaccination at enrollment

The 507 participants who were evaluated at the start of the study ranged from 2 to 83 years of age, with a mean ± SD of 21.4 ± 16.8 years, and 288 (56.8%) were male. The age and gender distributions are shown in Table 1. The mean time ± SD between the last vaccine dose and enrollment was 23.7 ± 1.7 months.

**Table 1:** Age and gender of the study participants at inclusion, and vaccine doses received in 2005.

Age and gender of participants in 2007				Number of vaccine doses received in 2005				
Age (years)	Male	Female	Total	2 doses	3 doses	4 doses	5 doses	Total
**2–5**	26 (5.1)	28 (5.5)	54 (10.7)	0 (0.0)	3 (5.6)	0 (0.0)	51 (94.4)	54 (100.0)
**6–15**	100 (19.7)	93 (18.3)	193 (38.1)	1 (0.5)	15 (7.8)	2 (1.0)	175 (90.7)	193 (100.0)
**16–40**	124 (24.5)	64 (12.6)	188 (37.1)	0 (0.0)	28 (14.9)	4 (2.1)	156 (83.0)	188 (100.0)
**41–60**	29 (5.7)	28 (5.5)	57 (11.2)	1 (1.8)	11 (19.3)	0 (0.0)	45 (78.9)	57 (100.0)
**>60**	9 (1.8)	6 (1.2)	15 (3.0)	1 (6.7)	2 (13.3)	0 (0.0)	12 (80.0)	15 (100.0)
**Total**	288 (56.8)	219 (43.2)	507 (100.0)	3 (0.6)	59 (11.6)	6 (1.2)	439 (86.6)	507 (100.0)

Data are expressed as numbers and (percentages) of participants

At enrollment, PEP had been given to 448 of the 507 participants (88.4%); 58 (11.4%) had received PrEP, and 1 (0.2%) had received a re-exposure PEP vaccination. In 2005, 340 subjects (78.0%) received rabies immunoglobulin. The number of vaccine doses administered in 2005 and participant age at inclusion are given in Table 1. To be eligible for the immunogenicity analysis, participants had to receive three vaccine doses for PrEP, five doses for PEP, or two booster doses for PEP following a suspected re-exposure. Most participants (439, 86.6%) had received five doses, six subjects (1.2%) had received four doses, 59 subjects (11.6%) received three doses, and only three subjects (0.6%) had received two doses. The number of doses does not exactly match the number and type of prophylaxis regimens given in 2005 because nine subjects who reported being given PEP had received fewer than five injections. Two of them had received only two vaccine doses, one received three doses and six received four doses. Those subjects were excluded from the analysis of both the boosted and non-boosted populations.

### Booster vaccination

Participants with antibody levels <0.5 IU/mL or EU/mL at inclusion or at one of the annual study visits were considered no longer seroconverted against rabies and were boosted. At enrollment in 2007, 2 years after vaccination, nine of the 507 participants (1.8%) had been boosted since receiving their PrEP or PEP regimens; six were given one or two booster injections, but the number of doses was not known for the three others. In 2008, 3 years after vaccination, 43 of the 461 participants with booster dose information (9.3%) had been boosted in the previous year. Forty of the 43 received one or two booster dose injections, one received five doses, and the number of doses was not known for two participants. In 2009, 4 years after vaccination, 14 of 428 remaining participants (3.3%) had received booster doses since their 2008 follow-up visit. Thirteen received one or two booster dose injections and one received three doses (Table 2).

**Table 2:** Booster history of all interviewed participants.

	Inclusion^a^	Follow-up 2008^b^	Follow-up 2009^c^
All interviewed participants, n	507	465	428
Booster dose received, n	507	461	428
No, n (%)	498 (98.2)	418 (90.7)	414 (96.7)
Yes, n (%)	9 (1.8)	43 (9.3)	14 (3.3)
Booster doses, n (%)	504	459	428
0 booster doses	498 (98.8)	418 (91.1)	414 (96.7)
1–2 booster doses	6 (1.2)	40 (8.7)	13 (3.0)
3–4 booster doses	0 (0.0)	0 (0.0)	1 (0.2)
5–6 booster doses	0 (0.0)	1 (0.2)	0 (0.0)

The subjects receiving booster vaccination included both those who received boosters due to a subsequent exposure and those who received boosters due to a serological result ≤0.5 IU/mL.

^a^ Boosted at inclusion (i.e., 2 years after vaccination)

^b^ Boosted 3 years after vaccination

^c^ Boosted 4 years after vaccination

### Possible rabies re-exposure

Twenty-six participants (5.1%) reported being bitten by an animal between vaccination in 2005 and enrollment in 2007; 21 of them (80.8%) had an RFFIT titer >0.5 IU/mL and 5 (19.2%) had a titer ≤0.5 IU/mL. Additionally, 9 (34.6%) had received a booster after the vaccination in 2005 and 17 (65.4%) had not. In the following year, 2007–2008, 34 participants (7.3%) were bitten; 32 (94.1%) had an RFFIT titer >0.5 IU/mL and 12 (35.3%) had received a booster after enrollment. Between their 2008 and 2009 study visits, 29 participants (6.8%) were bitten; 24 (82.8%) had an RFFIT titer >0.5 IU/mL, and 7 (24.1%) had received booster dose after enrollment. There was a total of 89 cases of re-exposure to rabies resulting from bites from rabid animals, mostly dogs (52 cases) but also bats, cats, and monkeys. No cases of rabies occurred among the study participants.

### RVNA persistence

**Fig 2:**
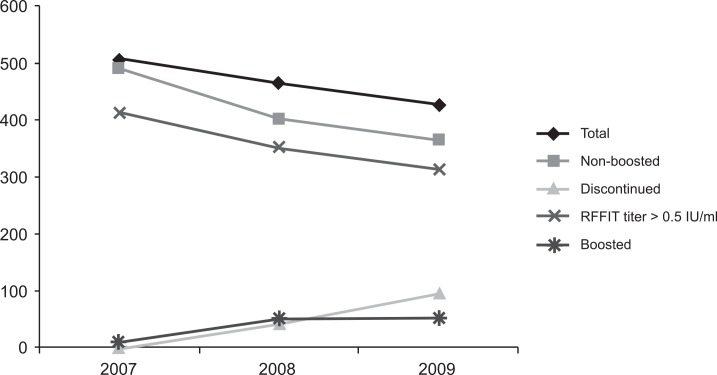
Cumulative totals of non-boosted, discontinued, seroconverted, and boosted study participants at each year from enrollment through 4 years after PEP or PrEP vaccination in 2005.

The serology results for both the non-boosted and boosted populations are shown in Table 3. In 2007, 2 years after vaccination, 413 (84.6%) of the 488 non-boosted participants had RFFIT RVNA titers >0.5 IU/mL. In 2008, three years after vaccination, 352 (88.0%) of the 400 evaluable, non-boosted participants had titers >0.5 IU/mL, while in 2009, four years after vaccination, 312 (85.7%) of the 364 evaluable non-boosted participants had RFFIT titers >0.5 IU/mL (Fig 2).

**Table 3:** Rabies virus-neutralizing antibody titers >0.5 IU/mL and GMTs at each follow up visit.

	2 years after vaccination (2007)	3 years after vaccination (2008)	4 years after vaccination (2009)
**Non-boosted population**			
Evaluable participants	488	400	364
RFFIT titers >0.5 IU/mL, n %: (95% CI)	413	352	312
	84.6 (81.1–87.7)	88.0 (84.4–91.0)	85.7 (81.7–89.1)
GMT (95% CI)	1.10 (1.00–1.20)	1.11 (1.03–1.20)	1.00 (0.94–1.07)
**Boosted population**			
Evaluable subjects	9	48	51
RFFIT titers >0.5 IU/mL, n %: (95% CI))	8	44	41
	88.9 (51.8–99.7)	91.7 (80.0–97.7)	80.4 (66.9–90.2)
GMT (95% CI)	1.94 (0.73–5.14)	2.50 (1.82–3.41)	1.33 (1.03–1.70)

Nine (1.8%) of the 507 participants had received rabies vaccine booster doses between vaccination in 2005 and enrollment in 2007 (Table 3). The time since the last vaccination was not known for five of them, but it was 0–6 months for one, 12–18 months for two, and 18–24 months for one. Additionally, 43 (9.3%) of the 465 participants present at their follow up visit in 2008 (3 years after vaccination) were boosted according to their titer measured during the 2007 campaign. The interval since the last vaccination was not known for 12 of the boosted participants, but was 0–6 months for 31, and 6–36 months for the remaining five. Fourteen (3.3%) of 428 participants received a booster at the 4-year follow up in 2009. In 2009, 41 (80.4%) of the 51 evaluable boosted participants had RFFIT titers >0.5 IU/mL; the mean GMT was 1.33 IU/mL. The interval from the last vaccination was 12–18 months for 28 of the participants, 18–24 months for six, 42–48 months for one, and was not known for 16.

### Age and gender differences in antibody persistence

**Fig 3:**
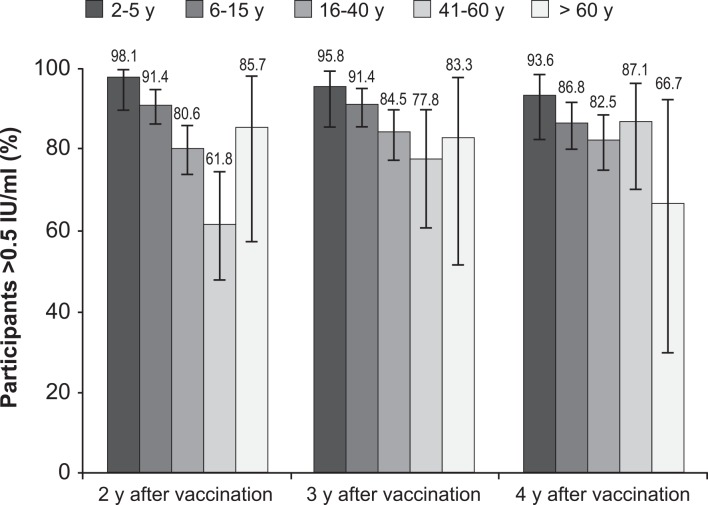
Age and seroconversion rates (RFFIT titer >0.5 IU/mL) of study participants at each follow up visit.

In the non-boosted population, GMTs (Table 4) were significantly higher in young participants 2–5 and 6–15 years of age and the proportion of subjects with RFFIT titers >0.5 IU/mL (Fig 3) was only slightly decreasing at each year of follow-up. In subjects aged 60 years or older, GMTs were lower although mostly >1 IU/mL, except for a drop between 2008 and 2009 where the seroconversion rate also decreased from 83.3% to 66.7%. However, the number of subjects was limited and the proportion of those with RFFIT titers >0.5 IU/mL was not significantly lower compared to the other age groups. In the 16–40 years age group, both the GMTs (around 1 IU/mL) and the proportion of individuals with RFFIT titers >0.5 IU/mL was stable over the 4 years of follow up. In the 41–60 years age group, the situation was far more contrasted with significantly lower GMTs (0.53 to 0.77 IU/mL) and proportion of subjects with RFFIT titers >0.5 IU/mL at inclusion and at the follow up visit in 2008, 3 years after vaccination (*P* <0.05, Fisher exact test) compared to the general study population. However, both values tended to increase over the years, thus suggesting that poor responders were progressively removed from the non-boosted population.

**Table 4:** GMTs (RFFIT) of the study participants by age group at inclusion and at 2, 3 and 4 years of follow-up after vaccination.

	GMTs (95% CI)		
	2007	2008	2009
Non-boosted population	1.10 (1.00–1.20)	1.11 (1.03–1.20)	1.00 (0.94–1.07)
Age at inclusion (years)			
2–5	2.03 (1.60–2.56)*	1.55 (1.26–1.91)*	1.34 (1.09–1.65)*
6–15	1.32 (1.15–1.51)*	1.23 (1.10–1.38)*	1.05 (0.95–1.16)*
16–40	0.96 (0.83–1.10)	1.00 (0.89–1.12)	0.91 (0.81–1.01)
41–60 years	0.53 (0.40–0.69)	0.70 (0.55–0.89)	0.77 (0.66–0.89)
> 60	1.02 (0.51–2.01)*	1.13 (0.66–1.93)*	0.93 (0.61–1.42)
*P*-value	**<**0.007*	<0.001*	<0.002*

*Statistically significant between-group difference of GMTs (Kruskal–Wallis test)

**Fig 4:**
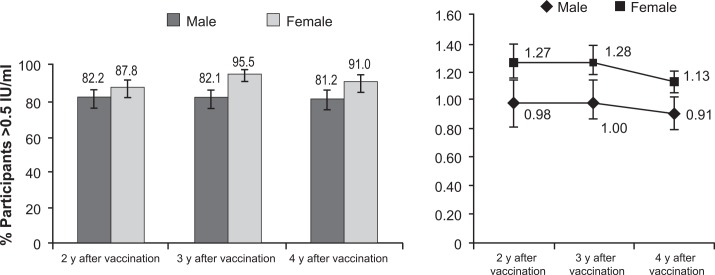
Seroconversion rates (titer >0.5 IU/mL) and GMTs of male and female participants at each year of follow up (RFFIT assay).

Males had lower seroconversion rates than females at each follow up visit, with significant differences observed in 2008 (*P* <0001) and 2009 (*P* <0.008, Chi squared test), 3 and 4 years after vaccination (Fig 4). Significant gender differences were also observed, with males having lower RFFIT GMTs than females at each year of follow-up. GMTs ranged from 1.27 [95% CI: 1.11–1.44] in 2007 to 1.13 [95% CI: 1.02–1.24] in 2009 in females and from 0.98 [95% CI: 0.87–1.11] to 0.91 [95% CI: 0.83–0.99] in males over the same years (Fig 4).

### Antibody persistence after pre- and post-exposure prophylaxis

**Fig 5:**
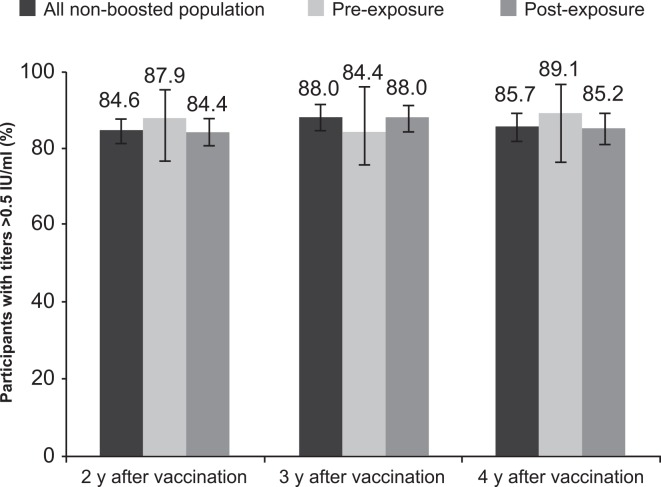
Seroconversion rates (RFFIT titer >0.5 IU/mL) of all non-boosted participants, and the non-boosted participants receiving PEP or PrEP vaccination in 2005.

**Fig 6:**
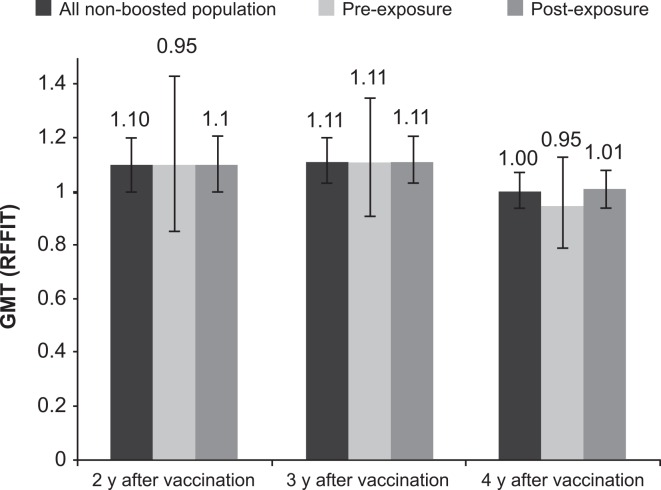
GMT (RFFIT) of all non-boosted participants, and the non-boosted participants receiving PEP or PrEP vaccination in 2005.

At each study visit, similar percentages of neutralizing antibody (RFFIT) titers >0.5 IU/mL were observed in the non-boosted participants who were given PrEP (3 vaccine doses, n = 58) in 2005 and in those receiving a PEP regimen (five vaccine doses, n = 448) at each study visit (Fig 5). Similar GMTs were also observed in the PrEP and PEP groups using the RFFIT assay, ranging from 1.0 to 1.1 IU/mL each year of follow up (Fig 6).

### RFFIT and FAVN assay results

All specimens collected in 2007 were retested with the FAVN assay to determine the correlations with the RFFIT assay and ELISA (Table 5). In both the non-boosted and boosted populations, strong correlations of the GMT values obtained with the FAVN and RFFIT assays were observed, *r* = 0.92 for the non-boosted (Table 5) and r = 0.99 for the boosted participants (Table 6). There was a good concordance of the seroconversion rates determined by FAVN (86.5%) and the RFFIT (84.6%) assays, with κ = 0.86. In the non-boosted population, the Pearson’s correlation coefficient for FAVN and ELISA was 0.83, (95% CI: 0.80–0.86). The result in the boosted population was similar (*r* = 0.95).

**Table 5:** Correlation between FAVN and RFFIT/ELISA techniques in the non-boosted population.

Two years after vaccination (in 2007)	
Non-boosted population, n	489
FAVN and RFFIT	
GMT	
FAVN	1.70
RFFIT	1.10
Pearson’s *r*	0.92
Subjects with titers >0.5 IU/ml	
FAVN	422 (86.5%)
RFFIT	413 (84.6%)
κ	0.86
FAVN and ELISA	
GMT	
FAVN	1.70
ELISA	1.01
Pearson’s *r*	0.83
Subjects with titers >0.5 IU/ml	
FAVN	422 (86.5%)
ELISA	387 (79.3%)
κ	0.61

**Table 6:** Correlation between FAVN and RFFIT/ELISA techniques in the boosted population.

Two years after vaccination (in 2007)	
Boosted population, n	9
FAVN and RFFIT	
GMT	
FAVN	2.88
RFFIT	1.94
Pearson’s *r*	0.99
Subjects with titers >0.5 IU/ml	
FAVN	8 (88.9%)
RFFIT	8 (88.9%)
κ	1.00
FAVN and ELISA	
GMT	
FAVN	2.88
ELISA	2.32
Pearson’s *r*	0.95
Subjects with titers >0.5 IU/ml	
FAVN	8 (88.9%)
ELISA	7 (77.8%)
κ	0.61

### RFFIT and ELISA results

There was a strong correlation between RFFIT and ELISA results (Pearson’s correlation coefficient) in the non-boosted population *r* = 0.82 at inclusion, which however progressively decreased over the years to 0.71 at the 1-year follow-up and 0.62 at 2-year follow-up. There was a good concordance of the proportion of titers >0.5 determined by the RFFIT (IU/mL) or the ELISA (EU/mL) assays; however the same trend was observed. The Kappa coefficient (κ) in the non-boosted population was 0.61 at inclusion, 0.54 at the 1-year follow-up and 0.42 at 2-year follow-up (Table 7). In summary, the strength of the association between RFFIT and ELISA decreased with time, as the GMTs obtained by RFFIT remained relatively unchanged over the duration of follow-up; and, unexpectedly, the ELISA values increased in the second and third years.

**Table 7:** Correlation between RFFIT and ELISA assay results in the non-boosted study population.

	Two years after vaccination (2007)	Three years after vaccination (2008)	Four years after vaccination (2009)
Non-boosted population (n)	489	402	365
GMT			
RFFIT	1.10	1.11	1.00
ELISA	1.01	1.31	1.39
Person’s *r* coefficient (95%CI)	0.82 (0.79–0.85)	0.71 (0.66–0.76)	0.62 (0.55–0.68)
Participants with titers >0.5 IU/mL [n (%)]			
RFFIT	413 (84.6)	351 (88.0)	312 (85.7)
ELISA	387 (79.3%)	347 (87.0%)	333 (91.5%)
Kappa coefficient	0.61	0.54	0.42

## Discussion

The primary objective of this study was to evaluate the persistence of RVNA following PrEP or PEP with PVRV as measured by sero-neutralization assays. Secondary objectives included describing the effect of booster doses on RVNA titers, estimating the incidence of clinical cases of rabies in the study population, and determining the correlation between the RFFIT, FAVN and ELISA rabies virus antibody assays. Adherence to the surveillance protocol was high, with 84% retention over 3 years of follow-up. Possible re-exposure, mainly from dogs, bats, monkeys, and cats was reported by 5–7% of participants each year. The low numbers of bat bites is probably evidence of effective preventive measures implemented in the region such as: the reduction of bat population using anticoagulants, improvement of dwelling places through the continuous supply of electric power and light (the absence of electric light is known to be associated with vampire bat attacks), and the protection of houses aimed at avoiding gaps in the walls, windows or doors [19,20].

### Persistence of RVNAs

Overall long-term persistence of RVNAs was good, with 85 to 88% of the non-boosted study population remaining seroconverted (RFFIT titer >0.5 IU/mL) over the 3 years of follow-up ending in 2009. The GMT of the population remained >1 IU/mL (twice the WHO-recommended threshold) at the end of follow-up. Persistence of RVNA following vaccination in 2005 was similar in participants given PrEP and those given PEP. These results are consistent with those reported in previous studies [21,22], and are discussed below in the context of routine PrEP vaccination. Our results are in accordance with other serological studies demonstrating that RVNA titers equal to or greater than 0.5 IU/mL, which is the WHO-recommended threshold of seroconversion, can persist for several years after administration of a complete vaccination series [23]. These results therefore highlight the need to maintain and intensify rabies PrEP and PEP.

There were gender- and age-related differences in RVNA persistence. Overall, females had significantly higher GMTs and higher seroconversion rates than males in 2008 and 2009. These results are in line with some previous reports [24,25], however a correlation between gender and immune response to rabies vaccine has not been established [26]. While some gender differences in this study were statistically significant, their clinical significance remains doubtful because the seroconversion rates remained above 80% and the GMTs above 0.90 IU/mL in both genders. Also, the persistence of RVNA, as measured by the seroconversion rate, was shorter in the population >60 years of age than in younger participants, but the difference was not significant, and GMTs decreased only slightly. Participants 16–40 years of age had lower immune responses than the other age groups, but the observed GMTs and seroconversion rates among that age group, at 0.91–1.0 IU/mL and 80.6–84.5%, respectively were similar to those observed in previous studies [22]. The GMT and seroconversion rate point values were lower in those 41–60 years of age than in the other age groups, and both increased over the duration of follow-up. These values may have been influenced by a relatively small sample size and broad 95% CIs. They also indicated the progressive removal of the poor responders from the study which mostly focused on the non-boosted population.

One of the limitations of the study is that only those subjects who responded well to the initial vaccination, i.e. remained seroconverted throughout follow up, were evaluated for antibody persistence. Subjects whose RFFIT antibody titer fell below or equal to 0.5 IU/mL were boosted and were excluded from the analysis to avoid any bias in evaluating antibody titers during subsequent follow up visits. Ideally, the analysis should have included all study subjects; however, it would have been both unethical and contrary to the design of our study (based on the recommendations presented in the leaflet of the rabies vaccine Verorab) not to vaccinate those with low antibody levels and expose them to the risk of rabies disease.

RVNA titers are generally measured by RFFIT [17] or FAVN, the gold standard assays recommended by the WHO [16]. Nevertheless, for additional analyses, an ELISA using rabies virus glycoprotein as antigen (Platelia Rabies II) is available [27,28]. Although this ELISA method does not measure human RVNA but all anti-glycoprotein G antibodies, it is easier and more rapid to perform. Rapid assays should be encouraged to facilitate diagnosis in rural settings lacking sophisticated techniques and qualified personnel. Increasingly discordant results were obtained with the RFFIT and ELISA assays from 2007 to 2009. The reason for these discordant results and for the decrease in correlation and concordance is not clear and deserves further investigation using well characterized proficiency panels. A previous comparison of these two assays found that the results of each corresponded closely except in samples with high RFFIT titers [27].

### PrEP in rabies endemic countries

This study and the dramatic events preceding it underscore the need for the health authorities in rabies enzootic countries to decide on the best timing for the introduction of routine rabies PrEP vaccination in affected areas even if regular titer checks and boosters, may not appear affordable for the developing economies. This introduction would also prevent the need for serotherapeutic treatment, a real advantage in developing countries where human or equine rabies immunoglobulins are scarce and expensive. Ideally, routine pre-exposure rabies vaccination should be included in the Expanded Program on Immunization (EPI) schedule, given concomitantly with other pediatric vaccines. Two studies have evaluated the concomitant administration of rabies and DTP vaccines in Vietnam, and a third evaluated the concomitant administration of rabies and Japanese encephalitis vaccines.

The first Vietnamese study in infants showed that PVRV can be administered concomitantly with DTP-IPV as 2 IM doses at 2 and 4 months of age and a booster dose 1 year later with satisfactory safety and immunogenicity results and with no interference between the 2 vaccines [29] [30] [31]. Similar findings were drawn from another study conducted in Vietnam where PVRV was co-administered with DTP-IPV as 3 ID or 2 IM injections. The study showed that there was no apparent interference between the 2 vaccines and confirmed that their co-administration was safe in infants and toddlers [32] [33]. Finally, a study conducted in Thailand confirmed that the co-administration of a purified chick embryo cell vaccine (PCECV) with Japanese encephalitis vaccine (JEV) is safe and confers satisfactory immune response without interference between the two vaccines [34].

These clinical studies strongly suggest that rabies vaccine may be co-administered with routine pediatric vaccines and support integration of rabies PrEP vaccination into the childhood immunization schedules of countries where rabies is enzootic. This would minimize the costs and practical difficulties associated with the introduction of rabies PrEP into routine immunization practice.

### Shortened PEP regimens

Shortened PEP vaccination regimens that require less than 1 month to complete are also particularly relevant in rural populations in rabies-endemic countries. They require fewer visits to the vaccination center, potentially resulting in better compliance. One option is abbreviated 4-dose IM schedule, which requires 2 weeks for completion. Preliminary data from studies conducted in Thailand [35] and India [36] suggest that a 1-week 4-4-4 intradermal (ID) PEP regimen is an alternative option to consider. An ongoing study in The Philippines (ClinicalTrials ID no. NCT01622062) is evaluating the 1-week 4-4-4 ID PEP regimen followed by a single-visit four-site ID booster vaccination at five years.

### Conclusions

The surveillance results obtained in this study should encourage health authorities in rabies-enzootic countries to investigate the best strategies and timing for introduction of routine rabies PrEP vaccination in affected areas. In terms of PEP regimens, our observation that a complete 3-dose PrEP schedule induced similar GMTs and similar percentages of vaccinees with RVNA titers >0.5 IU/mL compared to a complete 5-dose PEP schedule is in favor of abbreviated schedules.

## Supporting Information

S1 Checklist STROBE Checklist. (DOCX)
