# Supplementary material for: Persistence of Rabies Virus-Neutralizing Antibodies after Vaccination of Rural Population following Vampire Bat Rabies Outbreak in Brazil
Source: PLoS Negl Trop Dis. 2016 Sep 21;10(9):e0004920. doi: 10.1371/journal.pntd.0004920 (PMC5031405; doi:10.1371/journal.pntd.0004920)
Supplement: S1 Checklist — (DOCX) [file pntd.0004920.s001.docx]

STROBE Statement—checklist of items that should be included in reports of observational studies

|  | Item No. | Recommendation | Page  No. | Relevant text from manuscript |
| --- | --- | --- | --- | --- |
| **Title and abstract** | 1 | (*a*) Indicate the study’s design with a commonly used term in the title or the abstract | 2 | This prospective study was conducted in 2007 through 2009; study participants were followed-up annually. |
|  |  | (*b*) Provide in the abstract an informative and balanced summary of what was done and what was found | 2 | This study evaluated the persistence of rabies virus-neutralizing antibodies (RVNA) annually for 4 years post-vaccination. (…) The long-term RVNA persistence was good, with 85–88.0% of the non-boosted participants evaluated at each yearly follow-up visit remaining seroconverted. Similar RVNA persistence profiles were observed in participants originally given PEP or PrEP in 2005, and the GMT of the study population remained >1 IU/mL 4 years after vaccination. At the end of the study, 51 subjects (11.9% of the interviewed population) had received at least one dose of booster since their vaccination in 2005. |
| Introduction | | | |  |
| Background/rationale | 2 | Explain the scientific background and rationale for the investigation being reported | 5 | Following the rabies outbreak in 2005, more than 3,500 inhabitants of Augusto Correa received either post-exposure (PEP) or a pre-exposure (PrEP) prophylaxis. A few people were given booster vaccinations after possible rabies re-exposure, mostly following dog, bat, and monkey bites. |
| Objectives | 3 | State specific objectives, including any prespecified hypotheses | 6-7 (Methods) | The primary objective was to evaluate the persistence of RVNA following PrEP or PEP. Secondary objectives included describing RVNA titers following receipt of PVRV booster doses, estimating the incidence of clinical cases of rabies in the study population, and determining the correlation between the anti-rabies antibody titers obtained by the rapid fluorescent focus inhibition test (RFFIT) and a commercially available enzyme-linked immunosorbent assay (ELISA). |
| Methods | | | |  |
| Study design | 4 | Present key elements of study design early in the paper | 5-6 | This was a single-site, prospective epidemiological study designed to evaluate the persistence of RVNA in a population at risk of vampire bat rabies and who had previously received either PrEP or PEP regimens. The study also aimed at providing additional data on the need for booster vaccination against rabies. The results of 3 years of follow-up are presented here. |
| Setting | 5 | Describe the setting, locations, and relevant dates, including periods of recruitment, exposure, follow-up, and data collection | 6 | This prospective study was conducted in 2007 through 2009 at the Arai health unit (USF Arai 3) in Augusto Correa, to evaluate the persistence of RVNAs in those who had been vaccinated in 2005. |
| Participants | 6 | (*a*) *Cohort study*—Give the eligibility criteria, and the sources and methods of selection of participants. Describe methods of follow-up  *Case-control study*—Give the eligibility criteria, and the sources and methods of case ascertainment and control selection. Give the rationale for the choice of cases and controls  *Cross-sectional study*—Give the eligibility criteria, and the sources and methods of selection of participants | 6  8 (Statistical analysis) | Each study participant was followed-up annually for 3 years (in 2007, 2008 and 2009).(…) Anyone who had been vaccinated in 2005 was eligible to participate. (…)  Participants with RVNA titers ≤0.5 IU/mL or Equivalent Units (EU)/mL at enrolment or at subsequent annual visits received booster doses of PVRV.  The study populations included in the analysis comprised: 1) all the evaluable study participants in each follow-up year, 2) participants who received a booster dose of vaccine at enrollment or during a follow up year, and 3) participants who did not receive booster doses of vaccine either at study entry or in any follow-up year. |
|  |  | (*b*) *Cohort study*—For matched studies, give matching criteria and number of exposed and unexposed  *Case-control study*—For matched studies, give matching criteria and the number of controls per case |  | NA |
| Variables | 7 | Clearly define all outcomes, exposures, predictors, potential confounders, and effect modifiers. Give diagnostic criteria, if applicable | 8 | The primary study endpoint was the number and percentage of subjects with RVNA titers >0.5 IU/mL each year using the RFFIT assay. The study was designed to include analysis by gender and age group (i.e., 2–5, 6–17, 18–40, 41–60, and >60 years of age). The number and percentage of subjects with RVNA titers >0.5 EU/mL using the ELISA test was calculated in the overall study population for each of their follow up visits. |
| Data sources/ measurement | 8* | For each variable of interest, give sources of data and details of methods of assessment (measurement). Describe comparability of assessment methods if there is more than one group | 7 | Blood specimens (5 mL) were collected from each study participant at enrolment and at each of the three annual follow-up visits (when the patient came to the health center) for testing by RFFIT and enzyme-linked immunosorbent assay (ELISA). Blood serum specimens were divided into four 0.5 mL aliquots for testing. RVNA titers were measured by RFFIT (Pasteur virus strain in BHK21 cells) at the Centro de Controle de Zoonoses (CCZ) laboratory in São Paulo, Brazil. Ten percent of the specimens were randomly selected for RFFIT re-testing at Institut Pasteur laboratory in Paris, France, using a vampire bat virus strain. The concentration of rabies virus anti-glycoprotein antibodies (EU/mL) in each blood sample was determined by ELISA (Pasteur virus strain) at Institut Pasteur laboratory in Paris, France, using the Bio-Rad Platelia® assay as per the manufacturer’s instructions. The correlation between the RVNA titers measured by RFFIT and by fluorescent antibody virus neutralization (FAVN) assay (challenge virus strain in BHK21 cells) was estimated at the CCZ laboratory, São Paulo, Brazil, using the specimens collected in 2007, the first year of the study. |
| Bias | 9 | Describe any efforts to address potential sources of bias | 8  6  7 | Anyone who had been vaccinated in 2005 was eligible to participate.  Ten percent of the specimens were randomly selected for RFFIT re-testing at Institut Pasteur laboratory in Paris, France, using a vampire bat virus strain. |
| Study size | 10 | Explain how the study size was arrived at | 7-8 | The sample size calculation was based on an expected seroconversion rate of 90% at 5 years after the primary vaccination series. A total of 140 subjects were required to ensure a 95% precision for a two-sided CI of 5%. Assuming 30% of the participants would be lost to follow-up at 5 years after primary vaccination, a total of 200 subjects had to be included. However, to anticipate additional dropouts, subgroup analyses and insufficient sera for laboratory testing, the planned enrolment was 500 participants. |

Continued on next page

| Quantitative variables | 11 | Explain how quantitative variables were handled in the analyses. If applicable, describe which groupings were chosen and why | 7  8 | Seroconversion (RFFIT titer >0.5 IU/mL) rates and geometric mean antibody titers (GMTs) were calculated with their 95% confidence intervals (CIs).  The study was designed to include analysis by (…) age group (i.e., 2–5, 6–17, 18–40, 41–60, and >60 years of age).  Quantitative variables were analyzed using Student’s t-test or ANOVA (parametric data) and the Wilcoxon or Kruskal–Wallis test (nonparametric data). |
| --- | --- | --- | --- | --- |
| Statistical methods | 12 | (*a*) Describe all statistical methods, including those used to control for confounding | 7 | The immunogenicity analysis was descriptive; no hypotheses were tested. Seroconversion (RFFIT titer >0.5 IU/mL) rates and geometric mean antibody titers (GMTs) were calculated with their 95% confidence intervals (CIs). |
|  |  | (*b*) Describe any methods used to examine subgroups and interactions | 8 | Quantitative variables were analyzed using Student’s t-test or ANOVA (parametric data) and the Wilcoxon or Kruskal–Wallis test (nonparametric data). Qualitative variables were analyzed using the Chi square test (or Fisher exact test when frequencies were less than five for at least one category). The correlations of GMTs measured by two different assays were determined by Pearson’s correlation coefficient (r). The correlations between percentages of participants with titers >0.5 IU/mL or EU/mL measured by RFFIT, FAVN or ELISA were calculated using the Kappa coefficient (κ). |
|  |  | (*c*) Explain how missing data were addressed | 8 | Missing data were not replaced. |
|  |  | (*d*) *Cohort study*—If applicable, explain how loss to follow-up was addressed  *Case-control study*—If applicable, explain how matching of cases and controls was addressed  *Cross-sectional study*—If applicable, describe analytical methods taking account of sampling strategy | 7 | Assuming 30% of the participants would be lost to follow-up at 5 years after primary vaccination, a total of 200 subjects had to be included. However, to anticipate additional dropouts, subgroup analyses and insufficient sera for laboratory testing, the planned enrolment was 500 participants. |
|  |  | (*e*) Describe any sensitivity analyses |  | None |
| Results | | | | |
| Participants | 13* | (a) Report numbers of individuals at each stage of study—eg numbers potentially eligible, examined for eligibility, confirmed eligible, included in the study, completing follow-up, and analysed | 8-9 | A total of 509 participants were enrolled in 2007 (Figure 1). Two of the 509 participants were excluded because their vaccination dates could not be confirmed. Among the 507 participants included, 496 (97.8%) were immunized (either PEP or PrEP) in 2005, eight in 2004 and three in 2006. In 2008, 42 participants were discontinued and 465 (91.7%) returned for evaluation. In 2009, 53 of the 465 remaining participants were discontinued and 16 of the 42 subjects who had been discontinued in 2008 came back so that a total of 428 (84.4%) participants were present in 2009. |
|  |  | (b) Give reasons for non-participation at each stage | 9 | Among the 95 participants who did not complete the study or did not attend all of the visits, 91 (95.8%) were lost to follow-up and four died (one following an epileptic coma and three of different cancers). |
|  |  | (c) Consider use of a flow diagram | 29 | Figure 1. Participant disposition |
| Descriptive data | 14* | (a) Give characteristics of study participants (eg demographic, clinical, social) and information on exposures and potential confounders | 9 | The 507 participants who were evaluated at the start of the study ranged from 2 to 83 years of age, with a mean ± SD of 21.4 ± 16.8 years, and 288 (56.8%) were male. The age and gender distributions are shown in Table 1. |
|  |  | (b) Indicate number of participants with missing data for each variable of interest | 9 | In 2007, nine participants were excluded from analysis because they had not received a complete PEP schedule (i.e., <5 vaccine doses). Four additional participants were excluded from analysis in 2008 because of missing data (no booster dose information). |
|  |  | (c) *Cohort study*—Summarise follow-up time (eg, average and total amount) | 9 | The mean (±SD) follow-up duration was 22.6 ± 6.9 months (range 0.0–26.5 months). |
| Outcome data | 15* | *Cohort study*—Report numbers of outcome events or summary measures over time | 11 | In 2007, 2 years after vaccination, 413 (84.6%) of the 488 non-boosted participants had RFFIT RVNA titers >0.5 IU/mL. In 2008, three years after vaccination, 352 (88.0%) of the 400 evaluable, non-boosted participants had titers >0.5 IU/mL, while in 2009, four years after vaccination, 312 (85.7%) of the 364 evaluable non-boosted participants had RFFIT titers >0.5 IU/mL (Fig. 2). |
|  |  | *Case-control study—*Report numbers in each exposure category, or summary measures of exposure |  | NA |
|  |  | *Cross-sectional study—*Report numbers of outcome events or summary measures |  | NA |
| Main results | 16 | (*a*) Give unadjusted estimates and, if applicable, confounder-adjusted estimates and their precision (eg, 95% confidence interval). Make clear which confounders were adjusted for and why they were included | 11 | In 2007, 2 years after vaccination, 413 (84.6%) of the 488 non-boosted participants had RFFIT RVNA titers >0.5 IU/mL. In 2008, three years after vaccination, 352 (88.0%) of the 400 evaluable, non-boosted participants had titers >0.5 IU/mL, while in 2009, four years after vaccination, 312 (85.7%) of the 364 evaluable non-boosted participants had RFFIT titers >0.5 IU/mL (Fig. 2). |
|  |  | (*b*) Report category boundaries when continuous variables were categorized |  | NA |
|  |  | (*c*) If relevant, consider translating estimates of relative risk into absolute risk for a meaningful time period |  | NA |

Continued on next page

| Other analyses | 17 | Report other analyses done—eg analyses of subgroups and interactions, and sensitivity analyses | 11-12  12-13  13 | In the non-boosted population, GMTs (Table 4) were significantly higher in young participants 2–5 and 6–15 years of age and the proportion of subjects with RFFIT titers >0.5 IU/mL (Figure 3) was only slightly decreasing at each year of follow-up. In subjects aged 60 years or older, GMTs were lower although mostly >1 IU/mL, except for a drop between 2008 and 2009 where the seroconversion rate also decreased from 83.3% to 66.7%. However, the number of subjects was limited and the proportion of those with RFFIT titers >0.5 IU/mL was not significantly lower compared to the other age groups. In the 16–40 years age group, both the GMTs (around 1 IU/mL) and the proportion of individuals with RFFIT titers >0.5 IU/mL was stable over the 4 years of follow up. In the 41–60 years age group, the situation was far more contrasted with significantly lower GMTs (0.53 to 0.77 IU/mL) and proportion of subjects with RFFIT titers >0.5 IU/mL at inclusion and at the follow up visit in 2008, 3 years after vaccination (P <0.05, Fisher exact test) compared to the general study population. However, both values tended to increase over the years, thus suggesting that poor responders were progressively removed from the non-boosted population.  Males had lower seroconversion rates than females at each follow up visit, with significant differences observed in 2008 (P <0001) and 2009 (P <0.008, Chi squared test), 3 and 4 years after vaccination (Figure 4a). Significant gender differences were also observed, with males having lower RFFIT GMTs than females at each year of follow-up. GMTs ranged from 1.27 [95% CI: 1.11–1.44] in 2007 to 1.13 [95% CI: 1.02–1.24] in 2009 in females and from 0.98 [95% CI: 0.87–1.11] to 0.91 [95% CI: 0.83–0.99] in males over the same years (Figure 4b).  In both the non-boosted and boosted populations, strong correlations of the GMT values obtained with the FAVN and RFFIT assays were observed, r = 0.92 for the non-boosted (Table 5) and r = 0.99 for the boosted participants.  There was a strong correlation between RFFIT and ELISA results (Pearson’s correlation coefficient) in the non-boosted population r = 0.82 at inclusion, which however progressively decreased over the years to 0.71 at the 1-year follow-up and 0.62 at 2-year follow-up. |
| --- | --- | --- | --- | --- |
| Discussion | | | | |
| Key results | 18 | Summarise key results with reference to study objectives | 14 | Overall long-term persistence of RVNAs was good, with 85 to 88% of the non-boosted study population remaining seroconverted (RFFIT titer >0.5 IU/mL) over the 3 years of follow-up ending in 2009. The GMT of the population remained >1 IU/mL (twice the WHO-recommended threshold) at the end of follow-up. Persistence of RVNA following vaccination in 2005 was similar in participants given PrEP and those given PEP |
| Limitations | 19 | Discuss limitations of the study, taking into account sources of potential bias or imprecision. Discuss both direction and magnitude of any potential bias | 15 | One of the limitations of the study is that only those subjects who responded well to the initial vaccination, i.e. remained seroconverted throughout follow up, were evaluated for antibody persistence. Subjects whose RFFIT antibody titer fell below or equal to 0.5 IU/mL were boosted and were excluded from the analysis to avoid any bias in evaluating antibody titers during subsequent follow up visits. Ideally, the analysis should have included all study subjects; however, it would have been unethical not to vaccinate those with low antibody levels and expose them to the risk of rabies disease. |
| Interpretation | 20 | Give a cautious overall interpretation of results considering objectives, limitations, multiplicity of analyses, results from similar studies, and other relevant evidence | 14 | These results are consistent with those reported in previous studies [21,22], and are discussed below in the context of routine PrEP vaccination. Our results are in accordance with other serological studies demonstrating that RVNA titers equal to or greater than 0.5 IU/mL, which is the WHO-recommended threshold of seroconversion, can persist for several years after administration of a complete vaccination series [23]. These results therefore highlight the need to maintain and intensify rabies PrEP and PEP. |
| Generalisability | 21 | Discuss the generalisability (external validity) of the study results | 17 (Conclusion) | The surveillance results obtained in this study should encourage health authorities in rabies-enzootic countries to investigate the best strategies and timing for introduction of routine rabies PrEP vaccination in affected areas. In terms of PEP regimens, our observation that a complete 3-dose PrEP schedule induced similar GMTs and similar percentages of vaccinees with RVNA titers >0.5 IU/mL compared to a complete 5-dose PEP schedule is in favor of abbreviated schedules. |
| Other information | |  | | |
| Funding | 22 | Give the source of funding and the role of the funders for the present study and, if applicable, for the original study on which the present article is based | 18 | The study sponsor, Sanofi Pasteur, was involved in all stages of the study, including study design, data collection, analysis and interpretation, preparation of this article, and decision to submit the article for publication. |

*Give information separately for cases and controls in case-control studies and, if applicable, for exposed and unexposed groups in cohort and cross-sectional studies.

**Note:** An Explanation and Elaboration article discusses each checklist item and gives methodological background and published examples of transparent reporting. The STROBE checklist is best used in conjunction with this article (freely available on the Web sites of PLoS Medicine at http://www.plosmedicine.org/, Annals of Internal Medicine at http://www.annals.org/, and Epidemiology at http://www.epidem.com/). Information on the STROBE Initiative is available at www.strobe-statement.org.
